# Explanatory factors of polydrug use in mid-late teens and the relevance of information sources: Correlational and configurational assessment in Tarragona (Spain)

**DOI:** 10.3934/publichealth.2024039

**Published:** 2024-06-18

**Authors:** Jorge de Andrés-Sánchez, Francesc Valls-Fonayet, Anna Sánchez-Aragón, Inma Pastor-Gosálbez, Angel Belzunegui-Eraso

**Affiliations:** 1 Social & Business Research Laboratory, Rovira i Virgili University, Av. Catalunya, 35, 43002 Tarragona, Spain; 2 School of Nursing, Rovira i Virgili University, Av. Catalunya, 35, 43002 Tarragona, Spain

**Keywords:** adolescence, substance use, polydrug use, correlational methods, fuzzy set qualitative comparative analysis

## Abstract

**Background:**

Substance use among adolescents is a public health problem, and the simultaneous use of multiple substances aggravates this problem. Although the facilitators of specific substance use in adolescents have been widely investigated, polydrug use is a less common topic. Likewise, the role that the origin of the information available to adolescents regarding substance use plays in relation to polydrug use is practically unexplored.

**Objectives:**

This work analyzed the relevance of the origin of the information sources available to adolescents regarding substance use, among which we distinguished those that were monitored (or supervised) by public agencies from those that were unmonitored (or unsupervised) in the consumption of more than one substance. As control variables, we considered three individual factors and four environmental factors. The relevance of these sources was analysed from a dual perspective: on the one hand, their statistical relevance was measured, and on the other hand, how they combined with the control variables was analysed to identify risk and risk-free profiles in substance poly-drug use.

**Methods:**

This paper utilized a sample of *N* = 573 adolescents aged ≥17 years. This sample was collected from a survey administered in the spring of 2023. We examined the impact of unmonitored information sources (peers, siblings, and the Internet) and supervised sources (school, parents, and media) on the combined consumption of alcohol, tobacco, and cannabis. Additionally, we took three individual factors (gender, early onset of alcohol, and tobacco use) and four environmental factors (parental control, alcohol, tobacco, and cannabis use among peers) into account as control variables. Initially, we conducted a regression analysis to adjust for the impact of these factors on polydrug use. Subsequently, we employed a fuzzy set qualitative analysis (fsQCA) to investigate how predictor factors combined with the formation of adolescent profiles associated with polydrug consumption and nonconsumption.

**Results:**

Unmonitored information sources were associated with a greater incidence of poly consumption, with ORs of 1.703 and p values of 0.004. However, the protective effects of regulated information sources remained ambiguous. Among the variables positively linked with of polydrug use, are female sex (*OR* = 1.329, *p* = 0.0076), early alcohol consumption (*OR* = 4.680, *p* < 0.0001), and early tobacco consumption (*OR* = 3.242, *p* < 0.001) were the most important. Peer drinking (*OR* = 1.556, *p* = 0.0187) and peer cannabis use (*OR* = 1.351, *p* = 0.0226) were also significantly correlated. The use of the fsQCA made it possible to identify the profiles of adolescents associated to polysubstance use and non-use. The conditions of the configurations that explained use were characterized by an early onset of the joint consumption of tobacco and alcohol. The profile of non-consuming adolescents is that of adolescents whose peers do not use tobacco or cannabis and who have parental control and monitored sources.

## 1. Introduction

### 1.1. General considerations

Adolescence is a keystone phase in the physical and mental development of individuals, making substance use a particularly concerning issue [1]. Etiologically, adolescents tend to have a greater inclination for exploration, experimentation, and increased impulsivity in their behaviors, which poses a greater risk for the initiation of drug use [2].

The three most commonly consumed substances by adolescents are alcohol, tobacco, and cannabis. This holds true not only in Spain, as indicated by the 2021 Survey on Drug Use in Secondary Education conducted by the Spanish Observatory of Drugs and Addictions [3],[4] but also in other countries, such as the USA [5],[6], Australia [7], and Switzerland [8]. Therefore, in this study, we focused on the consumption of these three substances, which share the common characteristic of having neurotoxic effects on cognitive and mental functions. They contribute to the development of personality disturbances [9], mental disorders, depression, and attention deficits [10], while also increasing the likelihood of the future use of more potent drugs due to the Gateway effect [11]. Therefore, they can lead to health, economic, and social problems later in life [12].

Furthermore, alcohol use may lead to risky and aggressive behaviors and accidents [13]. Tobacco use is associated with a higher prevalence of upper respiratory tract infections and respiratory system development problems in teenagers. Similarly, intensive cannabis use has been linked to conditions such as schizophrenia and psychosis [10],[14].

When discussing polysubstance use, we refer to the consumption of two or more drugs, whether legal or illegal, within the same period of time [15], but not necessarily at the same time [4]. In Spain, the OEDA (Observatorio Español de las Drogas y las Adicciones, Spanish acronym for Spanish Observatory on Drugs and Addictions) reported that 46.3% of young people aged 14 to 18 years experienced polydrug use at some time, while 21.6% had not consumed any psychoactive substances [3]. Alcohol is the most frequently used substance in polysubstance use patterns and is often accompanied by tobacco and cannabis [3],[7],[15]. Mixing substances is considered a particular risk to the safety and well-being of young people, especially due to their potentially unpredictable additive or interactive effects [15].

However, the analysis of the factors affecting drug consumption by teenagers is usually conducted on specific substances, and the issue of polydrug use is much less examined [7],[12]. Substance use is influenced by a multitude of variables, often with intricate interactions among them. These factors include a wide range of elements, including personality traits, parenting style, school engagement levels, peer influences, etc. [16]–[18]. Within this context, numerous studies emphasized the significance of health literacy in promoting healthier lifestyles [19],[20]. Nonetheless, one aspect that has received comparatively less attention is the impact of the information adolescents receive about substance use [21].

Several authors have observed that health literacy acts as a protective factor against substance use in young people and teenagers [22]–[24]. However, information campaigns conducted in educational institutions and through mass media channels do not always have significant effects [25]. Both Switzerland [26] and Spain [21] reported that increased information about substance use among adolescents was associated with increased rates of tobacco and cannabis consumption. This phenomenon is referred to as the “information paradox” [21] of substance use. In other words, having more information about drug use does not always seem to be a protective factor for drug use. One plausible explanation for this finding is that the effectiveness of health literacy depends on the credibility and reliability of the information sources [27],[28]. Thus, a further examination of the influence of information sources on adolescent substance use is required [21].

The literature identified six commonly recognized sources of the influence on health literacy in young individuals: school, parents, media, peers, siblings, and the internet [26]–[31]. In the case of the first three sources, information about substance use is either monitored or supervised by public agencies. On the other hand, messages about the consequences of substance consumption from peers, siblings, and the internet are either unmonitored or unsupervised by public organizations. Therefore, while the information obtained by minors from monitored sources must discourage substance use, this may not be the case for those who are not monitored.

The preceding paragraphs highlighted the issues that motivate this paper. This paper aims to analyse the relevance of whether the sources of information recognized by adolescents regarding substance use are either monitored or not monitored by public agencies in terms of the prevalence of polydrug use. This impact is controlled by various factors, such as early use of alcohol and environmental variables and the use of substances by peers, which are commonly considered in the literature [16]–[18].

This paper seeks to address the following research questions (RQs):

RQ1: What is the net effect of each explanatory factor on polydrug use, with a special focus on information variables? Thus, in this RQ, we quantify the effect of the input variables on polydrug use.

RQ2: How do explanatory factors combine to produce polydrug use and nonsubstance use? In other words, this second RQ tries to state the concern of mid- to late-adolescent profiles on polydrug consumption and profiles that are far from being at risk for that practice.

The study focuses on mid- to late-aged adolescents. Adolescence represents a phase in an individuals' lives that spans a few years but is marked by continuous and profound changes of various kinds: physical, psychosocial, neural, etc. [32]. Consequently, adolescence can be divided into three primary periods: early adolescence (11–13 years), characterized by the initial biological changes typical of puberty; middle adolescence (14–17 years), characterized by shifts in biological maturation and the heightened significance of peer relationships; and late adolescence (17–19 years), during which adolescents attain greater emotional stability and heightened social awareness and begin contemplating their life objectives [33].

Therefore, focusing the analysis of polydrug use on a specific stage of adolescence, as undertaken in substance use studies [34],[35], may prove insightful given the considerable variability in individual development across these stages. In our study, we concentrate on the analysis of poly-use in mid- to late-year adolescents, who, being older, are anticipated to exhibit a higher prevalence of poly-drug use than adolescents in earlier stages of adolescence.

### 1.2. Theoretical groundwork

The most common sources of health knowledge for adolescents recognized by prior research are school, parents, media, peers, siblings, and the internet [26]–[31]. Within these sources, we can differentiate two groups: those in which messages about substance use are overseen by public agencies and professional associations (i.e., the first three) and those in which the supervision of the information they provide is practically non-existent (i.e., peers, siblings, and the internet).

In Spain, information from schools, parents, and the media is closely regulated and supervised, with a focus on discouraging substance use by highlighting the associated risks and harms. Although some studies recognize that adolescents are deterred from drug use when they perceive potential dangers [36],[37], information and risk perception do not always coincide as protective factors.

Educational institutions play a crucial role in imparting knowledge to young individuals. They serve as the fundamental cornerstone of youth education, with health literacy being a key component [29]. Education's primary objective is to promote the health and well-being of young people, and health literacy significantly contributes to this goal. In Tarragona, programs aimed at preventing substance abuse in schools are closely monitored by public authorities and healthcare professionals [38].

Parents have a significant responsibility for their children's welfare, as stipulated by international and national laws. This responsibility includes offering accurate information about the consequences of drug use. Spanish law even allows for the loss of custody in cases where parental behavior jeopardizes a child's well-being due to issues such as substance abuse [39].

Conventional media outlets, which are governed by strict legislation and ethical guidelines, serve as a platform to disseminate information about the risks associated with drug use. There are stringent bans on tobacco and alcohol advertising and sponsorships at both the European and national levels (European Union Directives 89/552/EEC and 2003/33/EC). Public health authorities use these platforms to raise awareness about the dangers of tobacco, and the content is scrutinized for accuracy. The ethical code of the Spanish College of Journalists ensures the accuracy of information presented in the media, among other things [40].

Some information sources have limited supervision in their influence on adolescents, potentially encouraging substance use. Peer-, sibling-, and internet-based channels of information lack effective regulation from both a legal and administrative standpoint. While unmonitored information is not necessarily false, it often lacks context and demands that the recipients possess health literacy for proper interpretations [20].

Peers and siblings frequently prioritize immediate pleasure and hedonism over long-term consequences. Their influence often revolves around themes such as social enjoyment, relaxation, and associating substance use with status and allure [41]–[43]. This can lead adolescents to underestimate the potential risks associated with substance use, compounded by their generally reduced perception of these risks [44].

The internet holds valuable health-related information and support networks, granting access to substance-use prevention resources [45] and online interventions involving professionals, peers, or a combination of both [46]–[48]. Nonetheless, the absence of control over this information can detrimentally affect health literacy and transform it into a source of health misinformation, especially concerning drug and tobacco consumption [49].

We considered three variables related to the environment or microsystem of the adolescent as control variables [16]–[18].

For the control variables associated with the individual characteristics of adolescents, we considered gender and early initiation of alcohol and cannabis use. Gender is a common control variable used in all epidemiological studies and is relevant to explain an adolescents' substance use habits. This includes physiological differences between men and women in their reactions to substances [50], as well as the tendencies that men and women may have regarding their consumption patterns. In this regard, there is no singular pattern in the literature, as it depends on the cultural context of the study and the prevailing gender roles [51].

The early onset of substance use is often established at ages up to 15 years [52] and is commonly associated with a continued or increased consumption of the same substances and with poly drug use [53]. Thus, the early alcohol consumption is related to alcohol consumption at later ages [54], which is often more severe and problematic [55]–[58]. The same considerations can be made regarding tobacco, where the common denominator in the literature relates early contact with this substance to later, more intense tobacco consumption and addiction [59],[60]. Early alcohol consumption [61],[62] or an early combination of alcohol and tobacco [63],[64] has often been found to precede the use of new and usually illegal substances, with cannabis being the most common.

Among the environmental control variables, the first factor we considered was parental control. The permissive attitudes of parents toward substance use can increase the risk of their consumption during adolescence, while parental disapproval acts as a protective factor [65]–[72]. Although parental control discourages substance use, it is widely recognized that an authoritarian parenting style with rigid and arbitrary rules and a lack of support can facilitate substance use. Conversely, affectionate and democratic parents that allow adolescents to feel supported and respected provide a shield against drug use [73]–[75].

One of the central elements within a teenagers' immediate environment is the impact that peers exert on their substance consumption. This influence is supported by several studies [17],[76] that highlight how peers can shape subjective norms related to substance use. Numerous research studies have consistently identified a strong association between the consumption of alcohol, tobacco, or cannabis and their use by peers [77].

**Figure 1.**
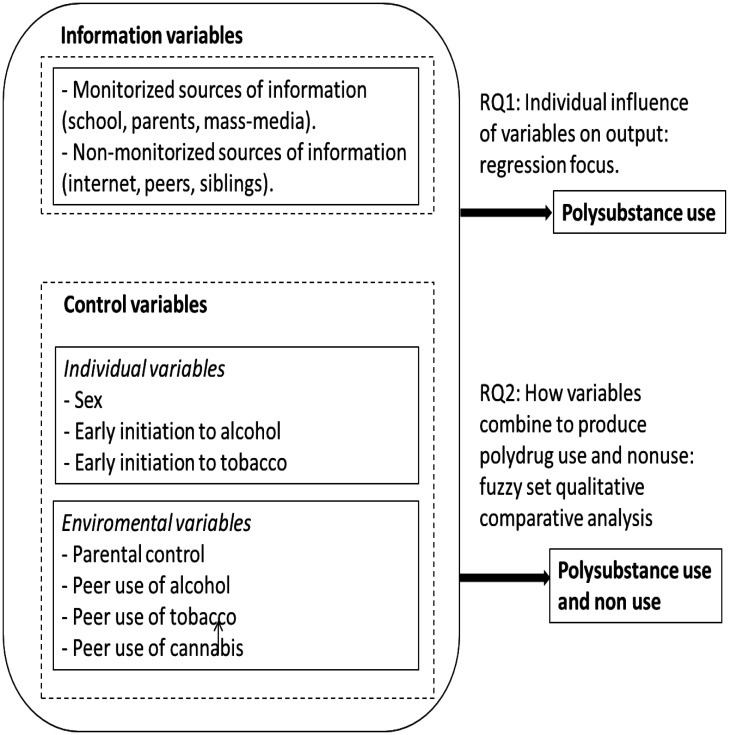
Groundwork used in this paper.

Thus, the framework with which we address the influence of information sources on polysubstance use is represented in Figure 1. Therefore, RQ1 will be addressed using such a framework that employs a correlational method such as ordered logistic regressions (OLR). The analysis of RQ2, which utilizes the same input-output variable framework as shown in Figure 1, aims to explore how the input variables combine in complex phenomena such as polydrug use. For this purpose, we will employ a fuzzy set qualitative comparative analysis (fsQCA).

## 2. Materials and methods

### 2.1. Materials

This study was conducted using a cross-sectional survey distributed among all the secondary schools and vocational educational centres in Tarragona, Spain, during the spring of 2023, in which adolescents aged at least 14 years responded. To carry out this research, we obtained permission and assistance from school principals with the help of social workers from the Tarragona City Council. The survey consisted of 66 questions, was completed online, and took approximately 15 to 25 minutes for each participant.

The total survey had 1307 responses, though we considered only those of mid- to late-year-old teens. Since we were interested in mid- and late-stage adolescents, we considered only the responses of adolescents who had either completed non-compulsory secondary school or who were enrolled in vocational training courses. The study utilized a final subsample of 573 responses from a larger population of no more than 5000 teenagers, thus ensuring a margin of error below 3% [48] and a 10% response rate over the overall objective population since the survey was distributed in all educational centres of Tarragona. Table 1 shows the characteristics of the sample of 573 mid- to late-year adolescents.

**Table 1.** Characteristics of the analysed sample of adolescents (*N* = 573).

**Category**	**Number of responses**	**Percentage**
*Gender*		
Females	251	43.80%
Males	311	54.28%
NA	11	1.92%
*Age*		
=17 years	462	80.63%
≥18 years	111	19.37%
*Mean* = 17.26, *SD* = 0.63		
*The adolescent lives with*		
at least 1 parent	521	90.92%
without parents	52	9.08%
*The adolescent was born*		
Spain	496	86.56%
Abroad	76	13.26%
NA	1	0.17%
*Parents of the adolescent were born*		
Both in Spain	496	86.56%
One in Spain	76	13.26%
None in Spain	61	10.65%

### 2.2. Variable measurement

The variables used in this paper were derived from the questions presented in Table 2, which were always answered on a discrete scale in natural numbers x = {0,1,2,…}.

The outcome displayed in Figure 1, namely polydrug use (PD_USE), quantifies the number of substances (alcohol, tobacco, and cannabis) consumed during the last 30 days. Initially, its values can be *x* = {0,1,2,3}.

As we pointed out in Figure 1 and section 1.2, the input variables are divided into two categories: those related to information and those used as controls to fit the adjusted significance of the information-related variables. Among the control variables, we differentiate between those related to the personal aspects of the adolescents and those related to their environment.

Regarding information sources about the substance use consequences, we differentiated between the monitored sources (i.e., school, parents, and mass media) and the unmonitored sources (i.e., peers, siblings, and the internet). Therefore, from Table 2, we find the following:

- Monitored sources (MONIT_S) = This value is initially obtained from the sum of the supervised information sources used by the adolescent, and we can take values in *x* = {0,1,2,3}.
- Nonmonitored sources (NON_MONIT_S) = This value is initially obtained from the sum of the unmonitored information sources used by the adolescent. Therefore, the possible values are *x* = {0,1,2,3}.

We can define the individual variables from Table 2 as follows:

- FEMALE = a dummy variable that takes 0 for boys and 1 for girls; this was directly obtained from a question asking for self-identified gender.
- Early initiation to alcohol (E_ALC) = A variable obtained from a question asking about the age of initiation to alcohol. It can take values in {1,2,...,5,6}, where 1 stands for “no initiation”, “2 = initiation at 15 years or after”, “3 = initiation at 14 years”,... and “6 = initiation at 11 years or before”.
- Early initiation to tobacco (E_TOB) = A variable obtained from a question asking for the age of initiation to tobacco. It can take values in {1,2,...,5,6}, and the interpretations of these values are analogous to those of E_ALC.

With regard to the environmental variables listed in Table 2:

- Parental control (PAR_CONTROL) = This variable is defined as the sum of the scores of the eight items listed in Q10 of Table 2. These items are based on the classical scale of parental monitoring in the questionnaire by Planet Youth [78], which has been extensively used in reports and in academic papers (see https://planetyouth.org/the-method/publications/). Notice that the items are answered on a four-point Likert scale that varies as follows: “1 = Does not apply at all to me”, “2 = Does not apply well to me”, “3 = Applies quite well to me”, and “4 = Applies very well to me”. Therefore, PAR_CONTROL can vary within *x* = {4,5,6,....,31,32}.
- Peer consumption of alcohol (P_ALC) = A question asked about the number of peers who consumed alcohol. A three-point Likert scale was used to indicate the number of peers who habitually consumed alcohol from “none” to “almost all”. Therefore, the possible values are *x* = {1,2,3}.
- Peer consumption of tobacco (P_TOB) was measured by asking about the number of peers who consumed alcohol. It was answered on the same Likert scale as P_ALC.
- Peer consumption of cannabis (P_CAN) was measured by asking about the number of peers who consumed alcohol. It was answered with the same scale as the two above variables.

To develop fsQCA, the scales for each variable shown in Table 2 were normalized and expressed in terms of a normalized value in [0, 1], which in a fsQCA setting was named the membership value. These membership values are displayed in Table 3. Likewise, for any variable *X*, its membership function is denoted as $m_{X}\;=\;{\left\{\frac{x}{m_{X}\left(x\right)}\right\}}_{x=0,1,\ldots }$.

With the exception of the variable linked to sex, the membership function for any input variable *X* is defined based on three points: the value from which the membership level is complete, *x_sup_* (i.e., $m_{X}\left(x_{sup}\right)\;=\;1$); the value from which this membership is null, *x_inf_*, *m_X_*(*x_inf_*) = 0; and the point at which there is maximum uncertainty (crossover point) *x_cross_*, *m_X_*(*x_cross_*) = 0.5. For the remaining values, *x*, a common procedure is determining membership through a linear interpolation [79].

The normalized values (or membership values) of all variables included in this analysis are displayed in Table 3.

**Table 2.** Summary of responses to questions linked with outcome and input items.

**Output items**			No	Yes	NA			
Did you use alcohol last 30 days?			274 (47.82)	280 (48.87)	19 (3.32)	
Did you use tobacco last 30 days?			438 (76.44)	120 (20.94)	15 (2.62)	
Did you use cannabis last 30 days?			506 (88.31)	56 (9.77)	11 (1.92)	

			0	1	2	3	NA	

PD_USE: Number of substances used last 30 days			246 (42.93)	179 (31.24)	76 (13.26)	36 (6.28)	36 (6.28)

**Input items**						

*Information items*						

My information about substance use come from...				No	Yes	NA

(Q1) school				144 (25.13)	389 (67.89)	40 (6.98)	
(Q2) parents/legal guardians				181 (31.59)	347 (60.56)	45 (7.85)
(Q3) mass media				202 (35.25)	329 (57.42)	42 (7.33)	
(Q4) internet				128 (22.34)	408 (71.20)	37 (6.46)	
(Q5) siblings				391 (44.50)	132 (47.29)	50 (8.20)	
(Q6) peers and friends				255 (68.24)	271 (23.04)	47 (8.73)	

			0	1	2	3	NA

MONIT_S: Monitored or supervised sources (Q1 + Q2 + Q3)			75 (13.09)	98 (17.10)	179 (31.24)	198 (34.55)	23 (4.01)

NON_MONIT_S: Nonmonitored or non supervised sources(Q4 + Q5 + Q6)			101 (17.63)	183 (31.94)	180 (31.41)	86 (15.01)	23 (4.01)

*Individual items*			

FEMALE (Q7): What is your sex?		(0)	(1)	NA			

		311 (54.28)	251 (43.80)	11 (1.92)				

Age of onset		(1)	(2)	(3)	(4)	(5)	(6)	NA

E_ALC: alcohol (Q8)		128 (22.34)	204 (35.60)	98 (17.10)	57 (9.95)	35 (6.11)	27 (4.71)	24 (4.19)

E_TOB: tobacco (Q9):		268 (46.77)	185 (32.29)	40 (6.98)	37 (6.46)	14 (2.44)	8 (1.40)	21 (3.66)

*Environmental items*								
PAR_CONT (Q10): Parental control		(1)	(2)	(3)	(4)	NA

PAR_CONT.1: My parents consider it important that my studies go well.		4 (0.70)	14 (2.44)	146 (25.48)	385 (67.19)	24 (4.19)
PAR_CONT.2: They establish clear rules about what I can do at home.		30 (5.24)	58 (10.12)	224 (39.09)	224 (39.09)	37 (6.46)
PAR_CONT.3: They establish clear rules about what I can do outside the house.		34 (5.93)	63 (10.99)	214 (37.35)	220 (38.39)	42 (7.33)
PAR_CONT.4: They establish clear rules about when I have to be home in the evening.		48 (8.38)	93 (16.23)	190 (33.16)	192 (33.51)	50 (8.73)
PAR_CONT.5: They know who I am with at night.		26 (4.54)	36 (6.28)	131 (22.86)	336 (58.64)	44 (7.68)
PAR_CONT.6: They know where I am at night.		18 (3.14)	29 (5.06)	116 (20.24)	370 (64.57)	40 (6.98)
PAR_CONT.7: They know my friends.		22 (3.84)	50 (8.73)	151 (26.35)	319 (55.67)	31 (5.41)
PAR_CONT.8: They know the parents of my friends.		79 (13.79)	98 (17.10)	190 (33.16)	158 (27.57)	48 (8.38)

		(0)	(1)	(2)	NA

P_ALC (Q11): How many of your friends do you think drink alcohol?		90 (15.71)	171 (29.84)	296 (51.66)	16 (2.79)
P_TOB (Q12): How many of your friends do you think use tobacco?		16 (2.79)	273 (47.64)	116 (20.24)	16 (2.79)
P_CAN (Q13): How many of your friends do you think use cannabis?		312 (54.45)	177 (30.89)	54 (9.42)	30 (5.24)

Notes: (a) In parentheses, the number of responses is presented as a percentage. (b) In sex (0) = Male and (1) = Female. (c) In Q8 and Q9, (1) = Never (2) = “≥15 years”. (3) = “14 years”, (4) = “13 years”, (5) = “12 years”, (6) = “≤11 years”, (d) In Q10: (1) It applies very poorly to me, (2) It applies poorly to me (3) It applies quite well to me, (4) It applies very well to me. (e) In Q11, Q12 and Q13, (0) = None, (1) = Some, (2) = Almost all.

**Table 3.** Membership functions of the variables used in this paper.

**Output variable**				**Membership function**
PD_USE				$m_{PD\_USE}\left(x\right)=\left\{\frac{0}{0},\frac{1}{0.5},\frac{2}{0.9},\frac{3}{1}\right\}$

**Input variables**	***x_inf_***	***x_cross_***	***x_sup_***	**Membership function**

MONIT_S	1	2	3	$m_{MONIT\_S}\left(x\right)=\left\{\frac{0}{0},\frac{1}{0},\frac{2}{0.5},\frac{3}{1}\right\}$
NON_MONIT_S	0	1	3	$m_{NON\_MONIT\_S}\left(x\right)=\left\{\frac{0}{0},\frac{1}{0.5},\frac{2}{0.75},\frac{3}{1}\right\}$
FEMALE	0	----	1	$m_{FEMALE}\left(x\right)=\left\{\frac{0}{0},\frac{1}{1}\right\}$
E_ALC	1	2	4	$m_{E\_ALC}\left(x\right)=\left\{\frac{1}{0},\frac{2}{0.5},\frac{3}{0.75},\frac{4}{1},\frac{5}{1},\frac{6}{1}\right\}$
E_TOB	0	0	2	$m_{E\_TOB}\left(x\right)=\left\{\frac{1}{0},\frac{2}{0.75},\frac{3}{1},\frac{4}{1},\frac{5}{1},\frac{6}{1}\right\}$
PAR_SUPP	20	26	30	$m_{PAR\_SUPP}\left(x\right)=\{\begin{array}{cc} 1 & \text{if }30\leq x\\ 0.5+\frac{x-26}{8} & \text{if }30\geq x>26\\ \frac{x-20}{12} & \text{if }26\geq x>20\\ 0 & \text{if }20\geq x \end{array}$
P_ALC	0	1	2	$m_{PEER\_ALC}\left(x\right)=\left\{\frac{0}{0},\frac{1}{0.5},\frac{2}{1}\right\}$
P_TOB	0	1	2	$m_{PEER\_TOB}\left(x\right)=\left\{\frac{0}{0},\frac{1}{0.5},\frac{2}{1}\right\}$
P_CAN	0	0	1	$m_{PEER\_CAN}\left(x\right)=\left\{\frac{0}{0},\frac{1}{1},\frac{2}{1}\right\}$

Note: With the exception of the membership functions of PD_USE and FEMALE, for the remaining variables, *x_inf_* = the 20th percentile of the variable, *x_cross_* = the 50th percentile and *x_sup_* = the 80th percentile.

### 2.3. Data analysis

This work sequentially applies OLR, which allows us to answer RQ1 and fsQCA, which is the technique employed to analyse RQ2. As a preliminary step, it should be noted that PAR_CONTROL is a scale composed of multiple items, so we will check its reliability by analysing Cronbach's alpha.

RQ1, which is linked with the measurement of the net effects of the information variables, is implemented while considering that the response variable is PD_USE, which can take the possible values {0,1,2,3}; however, the explanatory variables are quantified through their normalized values in Table 3. To answer RQ1, two models are fitted. In the first model, only the variables related to information are considered as explanatory variables. This allows us to obtain the unadjusted effects of the informational variables and their potential significance. Subsequently, we introduce the proposed individual and environmental control variables into the regression model, which allows us to assess their impact, which is of interest in itself, and the corrected net impact of the informational variables.

It is impossible to collect all the variables that can influence polysubstance use by adolescents; therefore, the number of variables must be limited to a manageable final number. McFadden's pseudo R2 can be very useful to measure the quality of fit of the overall model, with values above 20% considered an excellent fit in this regard [80].

RQ2 is answered by applying fsQCA with the software fsqca 3.1. In complex phenomena such as substance use, a given response can result from more than one pathway. In such circumstances, fsQCA is an appropriate method [81] because it is case-oriented. This technique allows us to measure the degree of membership of each case in the set of attributes and the outcome set by using a fuzzy set union and intersection operators [82]. Thus, fsQCA does not provide coefficients to quantify the influence of explanatory factors on the explained variable, but instead identifies various configurations in which the input variables combine to produce an output [79].

Notice that although fsQCA is a technique that involves data analysis, its name contains “qualitative.” One reason is that the main result, namely the configurations, does not contain coefficients. For example, a possible configuration for PD_USE could be the “presence of early use of tobacco”, the “early use of alcohol”, and the “absence of parental control.” However, there are no weighting coefficients in the conditions (the presence or absence of factors) in this possible profile. On the other hand, as a research approach, the foundations of a qualitative comparative analysis are explicitly rooted in qualitative, case-oriented research approaches in the social sciences, particularly in the understanding of causation as multiple and configurational, in terms of combinations of conditions, and in the conceptualization of populations as types of cases [83].

Although fsQCA may share certain similarities with structural equation modelling, it is a distinct technique that is well suited for our purposes. Modelling relationships with structural equations involving multiple interactions necessitates a prebuilt theory explaining why one variable interacts with another as either an input or as an output. In contrast, fsQCA can be used in a fully exploratory manner without the need to hypothesize about interactions among the input variables [84].

Moreover, while structural equation modelling ultimately yields measures of the importance of each variable to achieve an output, fsQCA identifies complete configurations of the input variables to produce the output [79]. Similarly, fsQCA is also appropriate when the relationships between variables are not symmetrical, although there are no restrictions on its use if they are symmetrical. This is evident in substance consumption, where profiles consistent with being consumers do not symmetrically align with those of non-consumers [85].

The use of fsQCA involves implementing the steps detailed in [80] and in the supplementary, with the ultimate goal of obtaining the necessary and sufficient conditions that lead to both polydrug use (PD_USE) and its absence, denoted as ~PD_USE, where “~” represents negation. In essence, the necessity analysis seeks to establish whether the presence or absence of an input variable is a necessary condition to produce the analysed output, which can be polydrug use or polydrug non-use in this paper. The sufficiency analysis establishes those pathways linked to the analysed output. These pathways can be interpreted in our context as profiles of mid- to late-aged adolescents of concern because they may tend to use substances or, conversely, profiles of low risk. These pathways or profiles are referred to as prime implicates in Boolean logic and as configurations, conditions, or recipes in the fsQCA literature.

In necessity and sufficiency analyses, the consistency (cons) and coverage (cov) measures of configurations are of a special interest since they quantify their explanatory capability. Consistency assesses the membership of a configuration in the outcome set and should have a value of cons > 0.75 to be considered sufficient. Coverage measures the proportion of the output set explained by a particular configuration and can be analogously interpreted to a coefficient of determination [79].

Additionally, while an input variable in a regression analysis can only be associated with an output with one sign (positive or negative), fsQCA allows for different signs in the influence of an input factor on the output variable. This property enables capturing all nuances of the influence of input variables on the studied outcome, as often this impact does not have a univocal sign [86]. For example, it is widely accepted that parental control tends to inhibit substance use, while permissive parenting styles can facilitate substance use [17],[72],[87]–[89]. However, some studies suggest that excessive parental control may facilitate substance use [17]. The use of fsQCA can reconcile both findings, which may appear contradictory, by showing that, in certain configurations associated with substance use, high parental control is a condition, while in others associated with non-use, it can also be a condition.

While less common than the use of correlational methods such as regression analysis, the use of fsQCA in quantitative studies in public health can be highly valuable in determining profiles for the analysed outcome [90]. For example, while demonstrating its usefulness in explaining the health status of older adults in Chinese provinces [91], this technique has also been used to identify adolescent profiles that facilitate and inhibit cannabis use [85]. In addition, it was applied to examine the characteristics of teleworkers who experienced stress and those who did not experience stress during the COVID-19 crisis [92].

## 3. Results

### 3.1. Descriptive statistics and analysis of RQ1 with correlational methods

Table 2 shows that the substance most consumed by adolescents was alcohol, with a 30-day prevalence rate close to 50% of the sample. Tobacco followed (with a prevalence of 21%), which was followed by cannabis (10%). The number of young people who did not consume any substance was 246 (43%). Only one substance was consumed by 179 (31.24%), two substances by 76 (13.26%), and three substances by 36 (6.28%).

**Figure 2.**
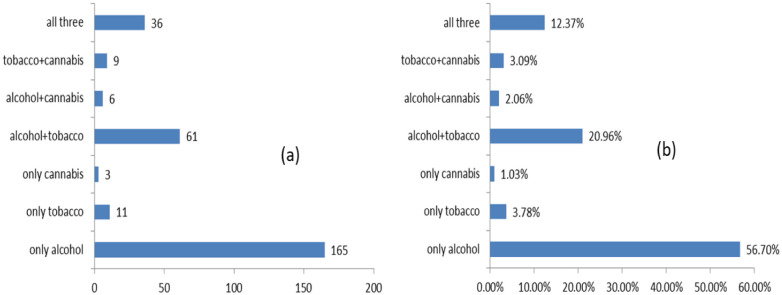
Alcohol, tobacco and cannabis consumption patterns for the last 30 days in our sample. Note: (a) shows the number of responses, and (b) shows the proportion of adolescents who consumed at least one substance for each pattern.

Figure 2 shows that the most individually consumed substance was alcohol (176 people, 56.70% of young people admitted to consuming a substance). Tobacco is rarely consumed individually (only 3.78% of consumers). However, 61 young people (20.96%) consumed alcohol. Cannabis is rarely consumed individually (by 3 people) or with a single substance (6 instances with alcohol and 9 with tobacco); most consumers indicated that they have also consumed both tobacco and alcohol in the last 30 days (36 people, 12.37% of consumers of at least one substance).

The most reported sources of information about the consequences of drug consumption for the respondents were the internet (71.20%) and school (67.89%). The least recognized sources were siblings (47.29%) and peers (23.04%). Thus, while most of the respondents acknowledged obtaining information from two or three monitored sources (31.24% and 34.55%, respectively), information from unsupervised sources was significantly less common. The most frequent responses were from either one source (31.94%) or two sources (31.41%).

Table 4 presents the results of the OLR regressions. Prior to the analysis, we assessed the reliability of the 8-item scale related to parental controls, yielding a Cronbach's alpha of 0.863.

The significance of the model that included only information variables was noteworthy, as indicated by a likelihood ratio of 38.33 (*p* < 0.01). However, McFadden's R2 was relatively low (2.90%). In this context, it is important to highlight that both information variables significantly contributed to explaining PD_USE. Specifically, the number of regulated information sources was a protective factor (*OR* = 0.61, *p* = 0.0008), while the unmonitored sources (NON_MONIT_S) significantly promoted poly-consumption (*OR* = 2.17, *p* < 0.01).

Turning to the complete model, we observed that the adjusted model of the ordered logistic regression exhibited a strong fit with the data. McFadden's R2 stood at an impressive 25.25%, which, within the realm of OLR, should be considered excellent [81]. Furthermore, the likelihood ratio underscored the model's high significance, with a value of 335.59 (*p* < 0.01).

A regression analysis revealed that all of the individual variables were significantly associated with polydrug use. Females exhibited a greater inclination toward poly-substance use (*OR* = 1.33, *p* = 0.0076). Similarly, early alcohol use (*OR* = 4.68, *p* < 0.01) and early tobacco use (*OR* = 3.24, *p* < 0.01) were significantly positively related to polydrug use.

Among the environmental variables, peer use of alcohol (*OR* = 1.56, *p* = 0.0187) and cannabis (*OR* = 1.35, *p* = 0.0226) were positively related to the consumption of multiple metals. However, peer use of tobacco and parental control did not achieve a statistical significance.

**Table 4.** Results of the ordered logistic regression on the number of substances used by teenagers.

Model with only information variables				Model including all the variables			

Information	*OR*	*p* value	95% *CI*	Information	*OR*	*p* value	95% *CI*
MONIT_S	0.610	0.0008	0.457–0.815	MONIT_S	0.80	0.171	0.584–1.101
NON_MONIT_S	2.716	<0.0001	1.964–3.756	NON_MONIT_S	1.70	<0.01	1.186–2.448

Individual	*OR*	*p* value	95%*CI*	Individual	*OR*	*p* value	95%*CI*

FEMALE	---	---	---	FEMALE	1.33	0.0076	1.079–1.638
E_ALC	---	---	---	E_ALC	4.68	<0.01	3.127–7.004
E_TOB	---	---	---	E_TOB	3.24	<0.01	2.356–4.460

Microsystem	*OR*	*p* value	95%*CI*	Microsystem	*OR*	*p* value	95%*CI*

PAR_CONTR	---	---	---	PAR_CONTR	1.30	0.204	0.865–1.968
P_ALC	---	---	---	P_ALC	1.56	0.018	1.077–2.250
P_TOB	---	---	---	P_TOB	1.17	0.444	0.777–1.777
P_CAN	---	---	---	P_CAN	1.35	0.022	1.043–1.749

McFadden's *R^2^* = 2.90% Likelihood Ratio Test 38.33 (*p* < 0.01)				McFadden's *R^2^* = 25.25% Likelihood Ratio Test 335.59 (*p* < 0.01)			

For the information variables, although the standardized number of the monitored information sources had a negative correlation with polydrug use (*OR* = 0.80), and this effect was not statistically significant (*p* = 0.173). On the other hand, a greater number of unmonitored information sources was associated with a greater prevalence of poly-consumption (*OR* = 1.70, *p* < 0.01).

### 3.2. Analysis of RQ2 with fuzzy set qualitative comparative analysis

Table 5 shows the results of the necessity analysis. The presence of early alcohol use (cons = 0.892) and peer alcohol use (cons = 0.885) has been shown to be a condition closely approaching to be necessary for polydrug use; however, the absence of early use of tobacco is also close to being a necessary condition for the non-use of multiple substances. Likewise, it is noteworthy that the presence of parental controls is nearly a necessary condition for both drug use and nondrug use.

Table 6 shows the sufficient conditions for polydrug use. For PD_USE, the solutions have cons = 0.743 and cov = 0.578 and include 6 profiles or configurations.

In regard to the information factors, the absence of MONIT_S consistently acts as a facilitator of combined substance use in the configurations they were involved in, and the act of not resorting to unsupervised sources to obtain information about substance consumption also consistently acts as a facilitator.

**Table 5.** Necessity analysis of the simple conditions of poly drug use and nonuse.

**Project**	**Poly drug use**		**Non poly drug**	
	**consistency**	**coverage**	**consistency**	**coverage**
MONIT_S	0.682	0.400	0.663	0.718
NON_MONIT_S	0.762	0.461	0.601	0.670
FEMALE	0.442	0.354	0.436	0.645
E_ALC	0.890	0.576	0.497	0.593
E_TOB	0.620	0.730	0.176	0.383
PAR_CONTR	0.925	0.362	0.892	0.645
P_ALC	0.885	0.449	0.654	0.611
P_TOB	0.732	0.566	0.443	0.631
P_CAN	0.628	0.548	0.281	0.452
~MONIT_S	0.519	0.455	0.446	0.721
~NON_MONIT_S	0.456	0.382	0.517	0.800
~FEMALE	0.558	0.349	0.564	0.650
~E_ALC	0.371	0.286	0.644	0.911
~E_TOB	0.620	0.731	0.876	0.809
~PAR_CONTR	0.093	0.319	0.119	0.744
~P_ALC	0.233	0.268	0.410	0.869
~P_TOB	0.524	0.338	0.696	0.827
~P_CAN	0.372	0.219	0.719	0.780

The most relevant condition is the early use of alcohol, which is a core condition in 5 configurations. The presence of E_TOB, P_TOB, and P_CAN is a core condition in the three configurations. The presence of parental controls is a core condition in two patients, and P_ALC is a core condition in one patient. Likewise, being female is a core condition in three profiles, while being male is a core condition in one. Therefore, E_ALC, E_TOB, PAR_CONTR, P_ALC, P_TOB, and P_CAN are always present in the prime, which implicates in which they take part. In contrast, being female is in three configurations linked of polydrug use and in one configuration of non-use.

In terms of substance non-use, Table 7 reveals that the obtained solution is comprised of 10 configurations with cov = 0.549 and cons = 0.908. Regarding sources of information, the absence of NON_MONIT_S is a condition in four non-use configurations, albeit as a peripheral condition. Conversely, the presence of MONIT_S is a core condition in three non-use prime implicates, while its presence is a peripheral factor in two prime implicates.

Notably, the absence of tobacco use (both early and by peers) and P_CAN are the most frequently observed conditions in the configurations. Specifically, ~E_TOB is a core condition in 7 configurations, while ~P_TOB and ~P_CAN are core conditions in 9 non-use configurations. The absence of early alcohol use is a core condition in five configurations. Additionally, parental control is a core condition in three configurations and a peripheral condition in another four configurations. The absence of alcohol use by peers is a peripheral condition in four prime implicates. Consequently, these results unequivocally demonstrate that the absence of E_ALC, E_TOB, P_TOB, P_ALC, and P_CAN are conditions of polydrug use in several configurations and that the presence of PAR_CONTR is also a condition. In some cases, this effect is strong, such as in the absence of cannabis consumption by peers, while in other cases, such as P_ALC, it is smaller.

**Table 6.** Intermediate fsQCA solution for poly drug use.

**Project**	**1**	**2**	**3**	**4**	**5**	**6**
MONIT_S	⊗		⊗			
NON_MONIT_S		⊗				
FEMALE	⊗	•		•	•	
E_ALC	•	•	•	•	•	•
E_TOB	•		•	•		•
PAR_CONTR	•	•			•	•
P_ALC		•	•	•	•	•
P_TOB			•	•	•	•
P_CAN			•	•	•	•
Coverage	0.151	0.196	0.190	0.159	0.191	0.366
Consistency	0.749	0.716	0.852	0.847	0.730	0.815
Coverage	0.578					
Consistency	0.743					

Note: Solid circles “•” indicate the presence of a condition, crossed circles “⊗” indicate their absence, and blanks “do not care”. Large circles represent core conditions, and small circles represent peripheral conditions.

**Table 7.** Intermediate fsQCA solution for the absence of polydrug use.

	**1**	**2**	**3**	**4**	**5**	**6**	**7**	**8**	**9**	**10**
MONIT_S	•	•					•	•	•	•
NON_MONIT_S			⊗	⊗	⊗	⊗				⊗
FEMALE	⊗	⊗				⊗	•	⊗	⊗	•
E_ALC			⊗				⊗	⊗	⊗	⊗
E_TOB	⊗		⊗	⊗	⊗	⊗	⊗	⊗		
PAR_CONTR	•	•		•	•	•		•	•	•
P_ALC			⊗	⊗				⊗	⊗	
P_TOB		⊗	⊗	⊗	⊗	⊗	⊗	⊗	⊗	⊗
P_CAN		⊗	⊗	⊗	⊗	⊗	⊗	⊗	⊗	⊗
Coverage	0.276	0.305	0.138	0.146	0.193	0.124	0.126	0.150	0.101	0.058
Consistency	0.868	0.938	0.989	0.981	0.987	0.981	0.977	0.978	0.965	0.975
Coverage	0.549									
Consistency	0.908									

Note: Solid circles “•” indicate the presence of a condition, crossed circles “⊗” indicate their absence, and blanks “do not care”. Large circles represent core conditions, and small circles represent peripheral conditions.

Notably, gender plays a role in four configurations as a core condition. Being male is a consistent core condition for non-use across these four configurations. However, it also appears as a peripheral condition in three configurations, and in two of them, being female is a condition. In other words, the impact of the FEMALE on the absence of polydrug use was not consistent. In some configurations where gender acted as a condition, it contradicted the sign of the relationship obtained with the OLR.

## 4. Discussion

For research question 1 (RQ1), we investigated the net effect of each explanatory factor on polydrug use in the previous month, with a special focus on the information variables. We found that in the model that considered only variables related to information, both supervised and unsupervised information sources significantly influenced the number of substances consumed in the last 30 days. In the first case, they acted as inhibitors, and in the second case, they acted as facilitators. However, when we adjusted for the net effects by including individual and environmental control variables in the model, only the facilitating effect of the number of unmonitored sources consulted by the adolescent remained significant. Additionally, it should be emphasized that although the variables considered in the explanation of polysubstance use in the expanded regression model did not capture all the complexity of the phenomenon, the McFadden's pseudo *R^2^* value, exceeding 20%, suggests that the fit has been practically excellent in a logistic regression setting [81].

Research question 2 (RQ2) inquired about how explanatory factors combined to produce polydrug use and nonsubstance use. We found various combinations of factors that led to both a tendency toward polydrug use and its inhibition. However, polydrug use and nonsubstance use did not have symmetric antecedents. In general, the presence of monitored sources and the absence of unmonitored sources were more evident in the configurations that explained nonsubstance users. In the polysubstance non-use, the presence of regulated information sources is a condition in six profiles, and the absence of unregulated information sources is a condition in five configurations.

Supervised information sources, which are subject to regulation by laws and public authorities, emerged as reliable sources of knowledge regarding the harmful consequences of drug use. Consequently, they appeared to enhance an adolescents' health literacy, equipping them with the awareness needed to make informed decisions concerning their wellbeing. In contrast, operating without regulatory oversight, unsupervised sources present a concerning contrast. These sources have the potential to undermine health literacy, as they may disseminate inaccurate or misleading information, making it challenging for adolescents to discern the actual risks associated with drug use. Moreover, this result suggested that people who receive their information from unmonitored sources such as peers or siblings might also be receiving encouragement to use substances, and some of those sources might also be substance users. Peer groups are known to have a strong association with substance use among adolescents. Those inclined to use for a variety of reasons connect with like-minded persons who might provide encouragement as well as information. However, this would not be true of monitored sources.

We observed that although the regression model that considered only the informative variables was significant, the introduction of control variables greatly improved the quality of the fit. Therefore, since polydrug use has been studied much less extensively than individual substance use, the findings regarding control variables can also be commented upon.

Regarding individual variables, early initiation of alcohol and tobacco consumption had the greatest impact on the number of substances consumed when measured as net effects, as they had the highest odds ratios (ORs). The configurational analysis showed that the presence of both factors was necessary in several explanatory configurations for polydrug use, with the age of alcohol initiation participating in a larger number of configurations. In contrast, the absence of early initiation in tobacco was involved in more configurations than the absence of alcohol initiation in explaining nonsubstance use.

Therefore, our findings are consistent with previous studies that related early substance initiation to polydrug use [53]. These findings also align with the fact that early alcohol consumption is positively associated with later consumption [54], often leading to a heavier use [57],[58]. These findings are also in line with reports regarding tobacco, which indicated that early contact with this substance was linked to later and more intense smoking habits and dependence [59],[60]. Furthermore, our findings are congruent with research that suggested that early contact with alcohol and tobacco often precedes cannabis use [53],[61]–[64].

The fact that the regression analysis indicated that females have greater polydrug use and that fsQCA suggests that being female tends to be a condition in polydrug use configurations, while being male tends to be a condition in non-consumer configurations, which can be considered a relative surprise. According to most of the literature reviewed [61], although the influence of gender on the analysis of individual substance use is highly variable, men tend to be more likely to engage in polydrug use. However, it is also true that for specific substances such as tobacco, many studies focused on Spain have found a higher prevalence among women [3],[12],[85]; moreover, in a longitudinal study conducted in Central Catalonia, a higher prevalence of combined tobacco and cannabis use among females was observed in some years [4].

For the variables within the adolescent microsystem, adjustments with ordered logistic regression (OLR) indicated that both alcohol and cannabis use by peers are significant facilitators of polydrug consumption. However, tobacco use by peers did not manifest as statistically significant in this regard. On the other hand, although it does not detect contradictory patterns, using fsQCA tends to give more importance to the relationship between peers with tobacco than peers with alcohol in explaining both polydrug use and nonsubstance use. The presence of peer tobacco and cannabis use is a core condition in 3 out of the 6 identified configurations of multiple substance use; however, peer alcohol use only participates as a core condition in one configuration. In the prime implicates of non-use, the absence of peers who consume tobacco and cannabis is a core condition in 9 out of the 10 detected profiles. However, the absence of peers who consume alcohol, although a condition in 4 configurations, is a peripheral condition. In any case, the relevance of peer substance use patterns in explaining adolescent behavior aligns with the mainstream literature [2],[15],[41],[43],[66],[67],[76],[77].

We found that parental controls did not have a statistically significant impact on polydrug use. However, this null net effect does not imply that parental control is not a relevant factor in explaining polydrug use. The analysis of the solutions of multiple consumption and non-use reveals that the presence of parental controls is necessary in various configurations explaining both outcomes. Therefore, we can infer that parental control influences substance consumption both positively and negatively, resulting in a null net effect. Thus, fsQCA allows us to reconcile the fact that the permissive attitudes of parents toward substance use increases the risk of substance use during adolescence, that an authoritarian parenting style with rigid and arbitrary rules can facilitate substance use, and that affectionate and democratic parents inhibit drug use by adolescents [17].

It should be noted that fsQCA allows us to capture the asymmetric impact of different factors analysed in terms of polydrug use and non-use [79],[85] on adolescents' cannabis consumption. The presence of variables related to early substance initiation is the most prevalent condition in the prime explanatory profiles explaining consumption. In contrast, the absence of tobacco and cannabis use by peers prevails in configurations related to the absence of substance use. In other words, while conditions linked to early substance use have the greatest presence in explaining polydrug use, those related to peer drug use have the greatest presence in inducing the absence of consumption.

The work conducted here has several methodological implications that deserve to be highlighted. We have found that fsQCA and correlational methods are not rival methods, but rather complementary methods. With the OLR, we measured the average impact of each analysed variable on the prevalence of polydrug use in our sample. However, with fsQCA, under sufficient conditions, we identified the profiles of mid- and late-ten adolescents who were a cause for concern, as they showed a high consistency with being consumers of multiple substances, and likewise, the profiles of adolescents who should be of a low concern because their characteristics were compatible with nonsubstance use. There is no single pattern that characterized both consumers and non-consumers; instead, in both cases, the outcome has more than one possible pathway.

Similarly, fsQCA allows for identifying different associations between the input variables to produce an output without the need for a prior theory. This finding can serve as a foundation for the subsequent modelling of interactions between input variables via correlational methods such as structural equation modelling.

Furthermore, through the utilization of fsQCA, we were able to confirm that explanations for both substance consumption and its absence place a greater emphasis on the early initiation of substance use as opposed to peer behaviors. By employing fsQCA, we can further fine-tune this discovery and observe that this heightened influence is linked to its facilitation of polydrug use. Conversely, fsQCA revealed that the absence of peers who smoked tobacco and cannabis was a pivotal condition in comprehending nearly all profiles associated with the inhibition of substance consumption.

Additionally, fsQCA allows us to determine that despite parental controls not appearing to be significant in the correlational analysis, it does not mean that parental controls are not a relevant factor to explain an adolescents' relationship with multiple substance consumption. The lack of statistical significance can be attributed to the fact that a parenting style characterized by excessive monitoring can both inhibit and stimulate substance use. This will depend on the specific parenting style to which the adolescent is exposed.

Moreover, the strong influence of the early onset of substance use on multiple drug consumption detected by correlational methods and its decisive role as a condition in explanatory configurations of both poly use (as “present”) and nonuse (as “absent”) justify health authorities and educational institutions placing special emphasis on designing campaigns and interventions to prevent substance use during early adolescence.

## 5. Conclusions

In this study, we conducted a cross-sectional survey in Tarragona, Spain, to explore the relationship between sources of information about the consequences of substance use and the prevalence of polydrug use among middle and late adolescents. We achieved this by employing a combination of correlational and configurational methods. A logistic regression enabled us to assess the net effect of the monitored and unmonitored information sources on adolescents' prevalence of polydrug use, while considering the individual and environmental control factors. A fuzzy set qualitative comparative analysis allowed us to identify how supervised and unsupervised information sources, when combined with individual and environmental factors, contributed to the profiles of polydrug consumers and no consumers.

Concerning substance use patterns, although alcohol is the most commonly consumed single substance, tobacco is often used concurrently with alcohol, and cannabis users frequently engage in the use of both alcohol and cigarettes.

Our regression analysis revealed that a greater presence of unsupervised sources significantly facilitated the use of one or more substances. Conversely, a configurational analysis showed that employing a variety of supervised information sources is a recurring core condition among thd profiles of adolescents who do not use substances.

Furthermore, as we consider the future implications of our study, we must remain vigilant about the continually evolving landscape of information dissemination. The digital age has brought forth new challenges and opportunities, especially with the internet becoming a major source of information. As regulations concerning online information evolve and as the behaviors of adolescents continue to adapt, our study serves as a snapshot in time. Therefore, the insights gained from our research should be interpreted with caution.

Additionally, wer recognize the relevance of highlighting the perspectives that this work can offer for the use of fsQCA in analysing the factors influencing substance use among young people. The results obtained regarding the significance of parental monitoring in the profiles of substance users and nonusers may encourage future configurational analyses that also incorporate parental warmth. This approach could allow us to visualize how different parenting styles (authoritarian, authoritative, permissive, and uninvolved) interact with other conditions that are relevant factors in substance use and nonuse.

## 6. Limitations

The findings of the present study illuminate the role of the types of sources from which late adolescents acquire information about substance use in polydrug consumption. However, we must also acknowledge several limitations.

First, our outcomes reflect the number of substances consumed by adolescents in the last 30 days but do not capture the intensity of consumption. Future analyses could distinguish not only whether a substance is consumed but also the frequency and intensity with which it is used in combination with others, whether sporadically or habitually. Additionally, we analysed self-reported data, and it is likely that substance use was underreported, especially considering that the survey was conducted within a school setting.

Second, the study was conducted using a sample collected during the spring of 2023 from adolescents in Tarragona, a city located in Catalonia—an Autonomous Community of Spain. Tarragona, with a population of more than 125,000 inhabitants, and Reus, another city with more than 100,000 inhabitants, constitute the major population centers in the region known as Camp de Tarragona, totaling nearly 500,000 inhabitants. This area is characterized by an economy focused on the chemical industry and tourism services, accompanied by a significant migrant population from South America and the Maghreb [92]. This demographic profile is a common feature shared with other coastal regions in Spain, as well as numerous towns in the metropolitan areas of Barcelona. Therefore, the obtained results may be more easily extrapolated to similar sociodemographic environments than to other regions, such as inland areas of Spain, whose economy is based on the primary sector and has a substantially different proportion of the migrant population.

Although the results indicate that an adolescent's self-reported early substance use is significantly correlated with polyuse incidences, this finding should be approached with caution since only a longitudinal study could fully elucidate it.

## 7. Ethical issues

(1) All participants and their legal guardians were informed about the study and the procedure; (2) anonymity of the collected data was ensured at all times; (3) the study was conducted with the authorization and support of the Tarragona City Council through its Committee for Addiction Prevention and the Department of Education of the Generalitat de Catalunya; (5) the study was conducted in accordance with the Declaration of Helsinki and approved by the Ethics Committee of the University Rovira i Virgili (CEIPSA-2021-PDR-39); and (6) questionnaire completion was voluntary for the children, with prior authorization from the school principal and their legal guardians.

## Use of AI tools declaration

The authors declare they have not used Artificial Intelligence (AI) tools in the creation of this article.


