# Supplementary material for: Explanatory factors of polydrug use in mid-late teens and the relevance of information sources: Correlational and configurational assessment in Tarragona (Spain)
Source: AIMS Public Health. 2024 Jun 18;11(3):773–802. doi: 10.3934/publichealth.2024039 (PMC11474328; doi:10.3934/publichealth.2024039)
Supplement: Supplementary file 1 [file publichealth-11-03-039-s001.pdf]

AIMS Public Health

AIMS Public Health, 11(3): 773–802.
DOI: 10.3934/publichealth.2024039
Received: 15 November 2023
Revised: 27 February 2024
Accepted: 26 March 2024
Published: 18 June 2024

https://www.aimspress.com/journal/aimsph

Research article

Explanatory factors of polydrug use in mid-late teens and the relevance of information sources: Correlational and configurational assessment in Tarragona (Spain)

Jorge de Andrés-Sánchez[1], Francesc Valls-Fonayet[2], Anna Sánchez-Aragón[1], Inma Pastor-Gosálbez[1] and Angel Belzunegui-Eraso[1,2,*]

[1] Social & Business Research Laboratory, Rovira i Virgili University, Av. Catalunya, 35, 43002 Tarragona, Spain
[2] School of Nursing, Rovira i Virgili University, Av. Catalunya, 35, 43002 Tarragona, Spain

* **Correspondence:** Email: angel.belzunegui@urv.cat; Tel: +34650442609.

Supplementary

Steps in fuzzy set qualitative comparative analysis

Step 1: Membership function calibration. This step involves transforming the responses of incomes into a normalized value in [0,1], which was already performed in section 2.2 in Table 3. In this regard, while the presence of a variable (a condition in the terminology used in configurational analysis) X is quantified by its membership function as m_X, its absence or negation $\sim X$ is calculated as $m_{\sim X} = 1 - m_X$.

Step 2: Conduct a necessity analysis of the conditions associated with the presence and absence of poly-drug use. A variable is deemed a necessary condition if its necessity measure exceeds 0.9.

Step 3: Define the sufficient conditions by establishing the configurations to explain polysubstance use and nonuse- These configurations are also referred to as prime implicates or recipes and can be assimilated to polydrug use and nonuse. To formulate these configurations, we need to determine the Boolean functions as follows:

PD_USE = f(MONIT_S, NON_MONIT_S, FEMALE, E_ALC, E_TOB, PAR_CONTR, P_ALC, P_TOB, P_CAN)

~PD_USE = f(MONIT_S, NON_MONIT_S, FEMALE, E_ALC, E_TOB, PAR_CONTR, P_ALC, P_TOB, P_CAN)

Step 4: Following the approach outlined in [31] for analysing the various pathways that explanatory factors follow to produce polydrug use, we will employ an intermediate solution. This solution consists of a set of configurations that include both the core conditions (present in both intermediate and parsimonious solutions) and peripheral variables (found only in the configuration within the intermediate condition).

AIMS Press

© 2024 the Author(s), licensee AIMS Press. This is an open access article distributed under the terms of the Creative Commons Attribution License (http://creativecommons.org/licenses/by/4.0)
